# Why does mobile payment promote purchases? Revisiting the pain of paying, and understanding the implicit pleasure via selective attention

**DOI:** 10.1002/pchj.765

**Published:** 2024-05-16

**Authors:** Qingguo Ma, Yulin Tan, Yijin He, Lu Cheng, Manlin Wang

**Affiliations:** ^1^ School of Management Zhejiang University Hangzhou China; ^2^ Institute of Neural Management Sciences Zhejiang University of Technology Hangzhou China; ^3^ Chinese Academy of Science and Education Evaluation Hangzhou Dianzi University Hangzhou China; ^4^ Business & Tourism Institute Hangzhou Vocational & Technical College Hangzhou China

**Keywords:** eye tracking, mobile payment, pain of paying, pleasure of payment, purchase behavior

## Abstract

The past years have witnessed a phenomenal growth of the mobile payment market, but how mobile payment affects purchase behavior receives less attention from academics. Recent studies suggested that lower pain of paying may not fully clarify the relationship between mobile payment and increased purchases (i.e., mobile payment effect). The current research first introduced price level in Study 1 and demonstrated that the pain of paying served as an underlying mechanism only in the high‐price condition rather than the low‐price condition. As such, Study 2 was conducted in a low‐price context to address the uncovered mechanisms. We propose a new concept of “pleasure of payment” that is defined as an implicit and consumption‐related hedonic response based on the cue theory of consumption. By tracking spontaneous attention to positive attributes (i.e., benefits) of products, Study 2 demonstrated this implicit pleasure as a psychological mechanism for the mobile payment effect when the pain of paying was not at play. These findings have important implications for mobile payment in research and practice by identifying price level as a boundary condition for the role of pain of paying and understanding the positive downstream consequences of mobile payment usage on consumer psychology.

## INTRODUCTION

Mobile payment (e.g., Alipay, Apple Pay) is gradually replacing cash and card payments with the ubiquity of smartphones and the support of mobile technology, and it has even become the mainstream payment method in some countries. For instance, Price Waterhouse Coopers (2019) reported that the proportion of mobile payment users in China had reached 86% in 2019. The COVID‐19 pandemic has also promoted the growth of the mobile payment market, as contactless payments could avoid virus transmission (Goel et al., 2022; Shishah & Alhelaly, 2021). It is even predicted that the global mobile payment market will reach 6 trillion USD by 2027 (Yahoo, 2022). However, the extant literature on mobile payment gives most attention to the antecedents of mobile payment adoption (Dahlberg et al., 2015; Gupta & Dhingra, 2022), while relatively neglecting an interesting topic of how the use of mobile payment (i.e., after the adoption) inadvertently affects purchase decisions. As payment is a critical part of the daily consumption process, exploring the downstream impact of emerging mobile payment technologies on consumers' purchase behavior and the underlying psychological mechanisms can provide new insights for individuals and societies to manage consumption activities.

Previous research on payment methods and consumer behaviors paid the most attention to comparing credit cards with cash. They consistently found that credit cards increased spending (e.g., Chatterjee & Rose, 2012; Liu & Chou, 2020; Thomas et al., 2011), namely the credit card effect (Feinberg, 1986). Similarly, several studies introduced mobile payment and found that it also facilitated purchases (i.e., mobile payment effect) (Falk et al., 2016; Liu, Luo, & Zhang, 2021; Ma et al., 2021), and the two effects were attributed to lower pain of paying (i.e., “an immediate negative emotion when giving money or imagining to give money”; Zellermayer, 1996, p.2).

However, recent studies have doubted the role of pain of paying in explaining the increased spending by measuring subjective self‐reported pain (Boden et al., 2020; Liu & Dewitte, 2021) and by observing objective neural signals in the anterior insula (Banker et al., 2021) that was a brain region reflecting the experienced pain of paying (Mazar et al., 2016). In other words, the pain of paying fails to cover all the differences in purchase behavior for different payment methods, which implies twofold research gaps in the field of knowledge. On the one hand, the contradictory results on pain of paying suggest that there may be boundary conditions for its mediation. Previous neuroscience research has proven that price positively affects the pain of paying (Knutson et al., 2007; Mazar et al., 2016). Thus, the price may modulate the effect of mobile payment on pain of paying and may be the boundary condition for the mediating role of pain of paying, which has not yet been explored. On the other hand, there may be other mechanisms specific to mobile payment that can explain the increased purchases. Several studies attempted to examine whether the convenience (i.e., reduction in payment steps and omission of bill calculation) drove spending (Boden et al., 2020; Liu & Dewitte, 2021; Liu, Luo, & Zhang, 2021), but found mixed results. Given that features like convenience can lead to a better shopping experience (Cavalinhos et al., 2021), there is limited insight into the impact of such positive use of mobile payment on consumer psychology, which is an internal drive of behaviors.

To address the above research gaps, we first focus on the much‐discussed pain of paying in Study 1 by introducing price level (low vs. high) to explore whether price moderates the mediation effect of the pain of paying. Then, in Study 2, we focus on whether there are other psychological elements to elucidate the mobile payment effect when pain of paying fails to explain this effect (i.e., in the low‐price condition). We build on the cue theory of consumption (Laibson, 2001) to hypothesize that pleasure of payment, an implicit and consumption‐related hedonic response arising from the long‐term positive use of a payment method, may serve as a psychological mechanism. Due to the implicit nature of this pleasure, Study 2 used eye‐tracking to record consumers' attention to product benefits (i.e., tasty or healthy) as an external indicator of the pleasure of payment, which is based on mood‐congruity effect (Bodenschatz et al., 2018; Bower, 1981; Tamir & Robinson, 2007). Both studies compared cash and mobile payment due to the direct leap from cash to mobile payment and the low penetration of credit cards in China.

Our work leads to theoretical and practical contributions to mobile payment and consumer behaviors. First, this study extends our comprehension of the pain of paying by identifying price level as an important boundary condition for the mediating role of pain in the mobile payment effect. Second, this study extends extant literature on cashless payments by adding the pleasure of payment as a new psychological mechanism behind the mobile payment effect. Concretely, according to the cue theory of consumption, we clearly define the concept of “pleasure of payment,” which deepens the understanding of how the pleasure of mobile payment is born and how it drives purchase behavior. We creatively capture the implicit pleasure from spontaneous selective attention and provide eye‐tracking evidence for the pleasure of payment, which also enriches consumer neuroscience. Finally, we discuss the practical implications for individuals and businesses by offering suggestions to attenuate or enhance the pleasure of mobile payment. To the best of our knowledge, this is the first eye‐tracking study to explore the implicit pleasure induced by mobile payment through visual attention.

## THEORETICAL BACKGROUND AND HYPOTHESES

### Pain of paying

The credit card effect (Feinberg, 1986), which refers to the promoting effect of a credit card versus cash on spending, has been extensively demonstrated by researchers (Chatterjee & Rose, 2012; Liu & Chou, 2020; Park et al., 2021; Prelec & Simester, 2001; Raghubir & Srivastava, 2008; Soman, 2001; Thomas et al., 2011). Mobile payment, however, has received less research attention on the topic of purchase/spending behaviors. Limited studies have shown that mobile payment facilitates purchases measured by willingness to pay (WTP; Falk et al., 2016), purchase likelihood (Liu, Luo, & Zhang, 2021), and purchase intention (Wang et al., 2022). This phenomenon is called the “mobile payment effect” in the present study.

Pain of paying, first proposed by Zellermayer (1996) to describe an immediate negative emotion that consumers experience from the (anticipated) act of giving money, is a leading underlying mechanism of the credit card effect. That is, consumers feel less pain when paying with a credit card, thereby increasing purchases. This difference in pain comes from payment transparency (Soman, 2003) and payment coupling (Prelec & Loewenstein, 1998). Specifically, the process of swiping a credit card renders the outflow of money invisible and omits the step of checking amounts, so credit card payment is less transparent, which leads to lower pain of money loss (Soman, 2003). “Coupling” refers to the extent to which payment is temporally related to consumption. The “buy‐now‐pay‐now” nature of cash payment creates tight coupling, while the delayed payment of a credit card facilitates decoupling between consumption and payment, thus reducing the pain of paying when making decisions (Prelec & Loewenstein, 1998). Interestingly, when including a debit card in the comparison, no difference in spending was found between the credit and debit cards, and the two card payments both decreased the pain of paying compared to cash (Park et al., 2021; Shah et al., 2016). These findings reveal that payment transparency is probably the primary reason for the difference in pain among payment methods.

From the perspective of transparency, mobile payment is considered to be the least painful payment form. Extant research has documented that the process of giving money is also unseen due to the support of quick response (QR) code and near‐field communication (NFC) technology (de Luna et al., 2019; Manshad & Brannon, 2021). Furthermore, consumers often use smartphones and payment applications (e.g., Alipay, WeChat) for non‐payment tasks (e.g., chatting with others, and browsing social media) before or even at the time of making payment (Boden et al., 2020), which distracts consumers from the payment process and then weakens the distinctiveness of payment (Gafeeva et al., 2018; Pisani & Atalay, 2018), thereby further reducing the pain of money loss.

To verify the role of pain of paying, some studies measured consumers' perceived pain through scales and neuroscientific methods. Measured by the Likert scale (e.g., Boden et al., 2020; Liu & Chou, 2020; Shah et al., 2016; Wang et al., 2022) and facial emoticon scale (e.g., Liu & Dewitte, 2021; Park et al., 2021; Thomas et al., 2011), as shown in Supplementary Material A, consumers indeed reported higher pain and fewer purchases when using cash versus cashless payments. Moreover, Mazar et al. (2016) have demonstrated that the pain of paying is a real emotional experience represented by the activation of the anterior insula rather than a metaphorical concept. Based on this finding, the greater activation of the insula elicited by cash payment might imply higher pain compared to credit card and mobile payment (Ceravolo et al., 2019). However, recent studies found inconsistent results. Banker et al. (2021) observed no significant signal difference in the anterior insula between credit card and cash in a functional magnetic resonance imaging (fMRI) study, and thus the signals failed to predict differences in subsequent purchases. Also, two cross‐country studies both found no evidence supporting the mediating role of pain of paying in spending behaviors (e.g., WTP or basket value) when it comes to mobile payment versus cash or credit card (Boden et al., 2020; Liu & Dewitte, 2021). Collectively, pain of paying seems not to be the sole mediator, as it cannot explain the credit card effect and mobile payment effect in some contexts. There may be boundary conditions for its mediation, such as the price level we discussed below.

### Price level

Essentially, the pain of paying is directly related to the financial loss. Pain arises when consumers realize that they will lose a certain amount of money in a consumption activity (Reshadi & Fitzgerald, 2023). Thus, it seems reasonable that the pain of paying will increase with the amount a consumer has to pay. Some researchers have provided neural evidence. Knutson et al.'s (2007) fMRI study first found that excessive product prices activated the insula prior to the purchase decision. Since the insula has been shown to be associated with expected pain (Wager et al., 2013) and negative arousal (Büchel, 2000; Paulus et al., 2003), this study shows that paying too much money can lead to direct negative experiences. Further, Mazar et al. (2016) robustly revealed that “pain of paying” is a high‐order emotional pain characterized by the activation of the anterior insula and reported a positive correlation between price magnitude and activity signals in the anterior insula. That is, the extent of the perceived pain varies with the magnitude of the price consumers (anticipate to) pay.

While price has been demonstrated to be another important factor influencing the pain of paying, it is necessary to consider both price level and payment method to explore their combined impact on the pain. One study focused on choice overload behaviors (Shah, 2015, experiment 3) and found that when the price of a pen increased from $0.25 to $1.00, the effect of payment form (student card vs. cash) on choice overload changed from non‐significant to significant. Based on the results, we propose that price level may modulate the effect of payment method on pain of paying in a similar way. That is, mobile payment (vs. cash) may reduce perceived pain at a high‐price level rather than at a low‐price. Price level may also modulate the mediating role of pain in the mobile payment effect as a result. In terms of the mobile payment effect, we focus on the impact of mobile payment versus cash on purchase intention. Stated formally:

The effect of payment method (mobile payment vs. cash payment) on pain of paying is moderated by price level (low price vs. high price). That is, in a high‐price condition, mobile payment reduces pain of paying; in a low‐price condition, there is no difference in pain between cash and mobile payment.

The mediating role of pain of paying is moderated by price level (low price vs. high price). That is, in a high‐price condition, pain of paying mediates the mobile payment effect; in a low‐price condition, pain of paying does not act as the mediator.

### Pleasure of payment

The cue theory of consumption (Laibson, 2001) and its neurophysiological evidence have been extensively applied to study the importance of situational cues on habit‐forming behaviors and marketing (Aufegger et al., 2021; Ben‐David & Bos, 2021; Karyadi & Cyders, 2019). Therefore, this theory can help us to understand how the (long‐term) use of a payment method subliminally impacts consumers' psychology and purchase behavior.

The cue theory of consumption takes conditioned learning as a micro‐foundation. The core content is that repeated and positive pairings are needed between an environmental cue and consumption to establish a positive association. Once the association is successfully established, the presence of the current cue can trigger an anticipated hedonic response (i.e., implicit pleasure), which motivates individuals to engage in consumption behaviors (Laibson, 2001). At the neural level, the above process is conducted by the hedonic forecasting mechanism (HFM) that is closely related to the brain's reward system (Bernheim & Rangel, 2004). Concretely speaking, as experience accumulates, the HFM associates a cue with biochemical responses following an action (e.g., shopping, playing games); as a result, when individuals are exposed to the cue again, the HFM produces the corresponding anticipatory hedonic response, evoking a desire to pursue this pleasure. The cue‐triggered desire is essentially a sudden increase in decision utility and drives individuals to take action (Berridge & Aldridge, 2009). Moreover, people are sometimes aware of the importance of cues but are never able to realize that their behaviors are manipulated by cue‐triggered pleasure and desire (Dijksterhuis et al., 2006; Winkielman & Berridge, 2004).

Based on this theory, both mobile and cash payments could be environmental cues associated with consumption, as Chinese consumers have long‐term usage experiences with them (China Payment and Clearing Association, 2022). The key to differentiating mobile payment from cash payment is the valence of association and its strength, which depends on the overall consumption experience of each purchase made by a payment method. The consumption experience might be particularly positive for mobile payment due to the following three unique characteristics. The first is a fluent payment process. Since consumers always carry their smartphones, they just need to show their QR code to the merchants without entering a password (Liu, Wu, & Yu‐Buck, 2021); whereas for cash payment, consumers need to search for their wallets, take out the cash, count the amount, and finally hand it to the merchant. Mobile payment increases payment fluency and speeds up the processing at the point of sale by omitting many unnecessary steps. Since fluency itself has hedonic features (Reber et al., 2004), the increased fluency of mobile payment could bring more positive consumption experiences. The second is the positive perception of mobile devices. Smartphones, as the physical carrier of mobile payment (Cavalinhos et al., 2021), are an enjoyable object nowadays due to diversified entertainment applications, and consumers are becoming attached and even addicted to their phones (Demirci et al., 2015). When shopping with mobile payment, consumers' positive perception of smartphones also imperceptibly increases the hedonic value of shopping experiences, which is not available for cash payment. The third characteristic is the low pain of paying. The double‐entry mental account (Prelec & Loewenstein, 1998) indicates that the consumption entry records the net utility of consumption, which is equal to the utility of acquiring goods minus the disutility of payments. Therefore, the lower the pain of mobile payment versus cash, the higher the net utility of consumption, which implies a more satisfying consumption journey.

As such, the positive association between mobile payment and consumption is stronger than that of cash payment, and cash payment may even negatively associate with consumption if the monetary loss is too salient for some consumers. The successful establishment of an association is reflected in the sensitization of reward networks in the brain (Banker et al., 2021; Berridge & Aldridge, 2009), after which implicit pleasure is born as a new emotion and can be evoked by the payment cue. Consequently, we propose that mobile payment could elicit a greater pleasure than cash payment—in this way increasing motivation to spend and fueling more purchases. As the implicit pleasure is based on the long‐term positive use of a payment method, it is defined as “pleasure of payment” in the current study. Stated formally:

Compared to cash payment, the use of mobile payment increases the pleasure of payment.

The effect of payment method (mobile payment vs. cash payment) on purchase intention could be mediated by the pleasure of payment.

### Implicit pleasure and selective attention

Berridge and Winkielman (2003) suggested that individuals cannot accurately detect their unconscious emotions through self‐report, even if these emotions influence subsequent behaviors. The mood‐congruity effect, which is a particularly well‐known and robust effect in the social cognition literature, perhaps provides an operationalized measure of the implicit pleasure we propose.

Specifically speaking, Bower (1981) first introduced the mood‐congruity effect by positing that mood would enhance the salience of mood‐congruent materials for selective attention. Then, a considerable amount of follow‐up research confirmed that individuals in a certain mood state (positive or negative) selectively attend to information consistent with their mood (Becker & Leinenger, 2011; Tamir & Robinson, 2007; Wadlinger & Isaacowitz, 2006), directly in the form of sustained fixations in eye tracking (Blanco & Vazquez, 2021; Bodenschatz et al., 2018; Kellough et al., 2008; Sanchez et al., 2014). There are also some extensions to the mood‐congruency effect. For instance, studies showed that positive mood directed attention to rewarding information more generally (e.g., reward words; Tamir & Robinson, 2007; achievement, social, and primary reward; Raila et al., 2015) besides positive emotional information (e.g., happy faces), which extends the meaning of positive information. Furthermore, Bodenschatz et al. (2018) used eye‐tracking to find that implicit affect was a better predictor of sustained attention toward affect‐congruent information than explicit affect, and that this was an unintentional process of attention allocation. These studies have broadened the scope of selectivity effects.

Based on the above studies, when it comes to a shopping situation, consumers will spontaneously deploy greater attention to the positive information if mobile payment indeed elicits higher pleasure. We thus in turn tested the pleasure of payment by observing this kind of selective attention. In terms of the product information, Chatterjee and Rose (2012) broadly categorized it into positive attributes, which referred to the good aspects of products (e.g., tasty or healthy) and were termed as benefits, and negative attributes, which referred to the poor aspects (e.g., non‐tasty or unhealthy) and were termed as costs. Therefore, we focused on consumers' attention to product benefits. As for the specific metric of attention, we used the total time the eyes gazed at a given area (i.e., fixation duration, which is also labeled as total fixation time), as fixation duration is a measure of sustained attention and in‐depth processing that is often used in eye‐tracking research on mood‐congruity effect (Blanco & Vazquez, 2021; Isaacowitz et al., 2008; Raila et al., 2015; Sanchez & Vazquez, 2014; Tang et al., 2019).

**FIGURE 1:**
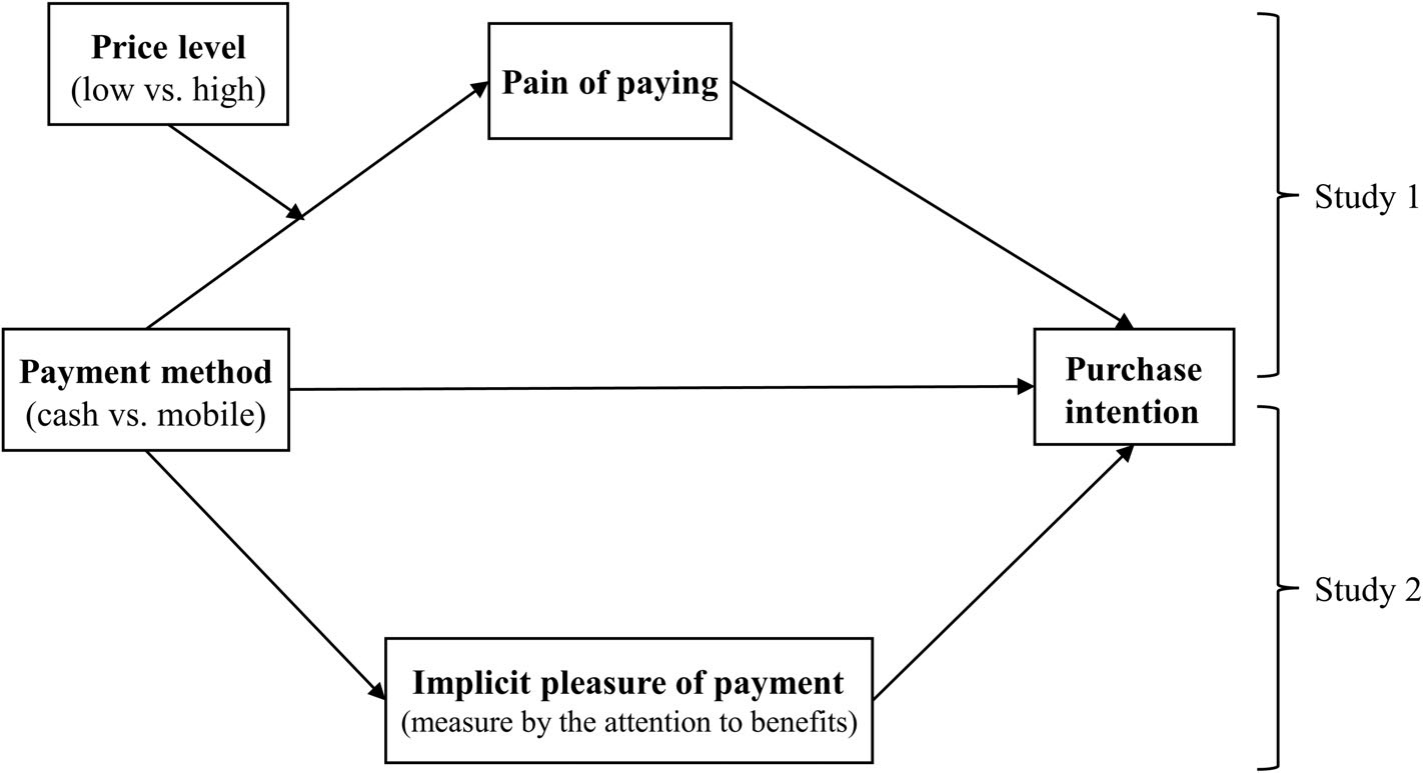
Research model.

Figure 1 shows the research model in this study.

## STUDY 1: ONLINE EXPERIMENT

### Participants and design

Study 1 was conducted online to test H1 and H2, with a 2 (payment method: mobile payment vs. cash payment) × 2 (price level: low price vs. high price) between‐subjects design. A total of 168 subjects were recruited from Credamo (https://www.credamo.com) in China with CNY 2.00 for participation. After excluding subjects with too short answering time (<250 s, average answering time = 560 s) and identical IP addresses, the final valid sample size was 160 (50% female; mean age = 28.20 years, *SD* = 1.30 years) that were randomly assigned to one of four conditions.

We focused on the purchase of food, which is a kind of fast‐moving consumer good (necessary and high‐demand) commonly found in stores (Sardana et al., 2018). The choice of food as a material is also similar to prior literature on payment methods (Boden et al., 2020; Liu & Dewitte, 2021; Park et al., 2021; Thomas et al., 2011). Fifteen imported food items were selected from eight categories (i.e., cookie, pastry, shrimp strip, potato chip, beverage, yogurt, candy, and chocolate; see Appendix A) as experimental products. We manipulated the payment method and price level. First, the payment method was primed in the form of a video (Ceravolo et al., 2019) and picture (Banker et al., 2021; Thomas et al., 2011). In detail, the cover story informed subjects which payment method (mobile payment or cash payment) was the only method accepted by the supermarket and showed a video of the corresponding payment process; the picture of the payment method was on the page that displayed a product and the scale of purchase intention. Second, in terms of the price level, a pretest (*n* = 44; 39% female; mean age = 23.07 years, *SD* = 1.34 years) was conducted to obtain two prices for each food category. We required subjects to write down a price that did not affect purchase decisions at all (filling in the maximum value if there is a range in mind) and a price that made decisions quite wavering (filling in the minimum value if there is a range in mind). The respective lower quartile values were used as the low price and the high price of each category in Study 1 (see Appendix A). Hence, for most individuals (i.e., 75% of subjects), the two values represent a low price and a just‐acceptable price (neither too low nor too high) for each food category, which somewhat corresponds to Shah's (2015) study.

### Procedure and measures

In the low‐price (high‐price) condition, subjects were told that all products were discounted by 60%–70% (10%–20%) due to trial operation during the opening. This background was to make the prices that were lower than the real retail prices more reasonable. In the mobile payment (cash payment) condition, subjects were told that the supermarket could only accept mobile payment (cash payment), and they watched a video showing one hand tapping out the Alipay QR code and then placing it in front of the scanning machine (one hand counting the amount of money and then handing it to the cashier). A total of 15 products were displayed in the experiment. On each webpage, we showed the picture and price of a product as well as the picture of payment method, and the subjects' purchase intention was assessed (1 = *totally unwilling*, 7 = *totally willing*; Nascimento et al., 2022). On the next page, subjects evaluated their pain of paying (e.g., “How painful would you feel to pay CNY 3.50 by smartphone/cash when considering purchase intention?”; 1 = *not at all painful*, 7 = *very painful*; Boden et al., 2020; Liu & Chou, 2020; Rick et al., 2008; Shah et al., 2016). Afterward, subjects were asked about their preference for each product (1 = *totally dislike*, 7 = *totally like*), the frequency of mobile shopping (e.g., “On average, how many times do you use smartphones for shopping in a day?”; Hou & Elliott, 2021) and demographics. The descriptive statistics for the mobile shopping frequency are shown in Supplementary Material B.

### Results

#### Pain of paying

**FIGURE 2:**
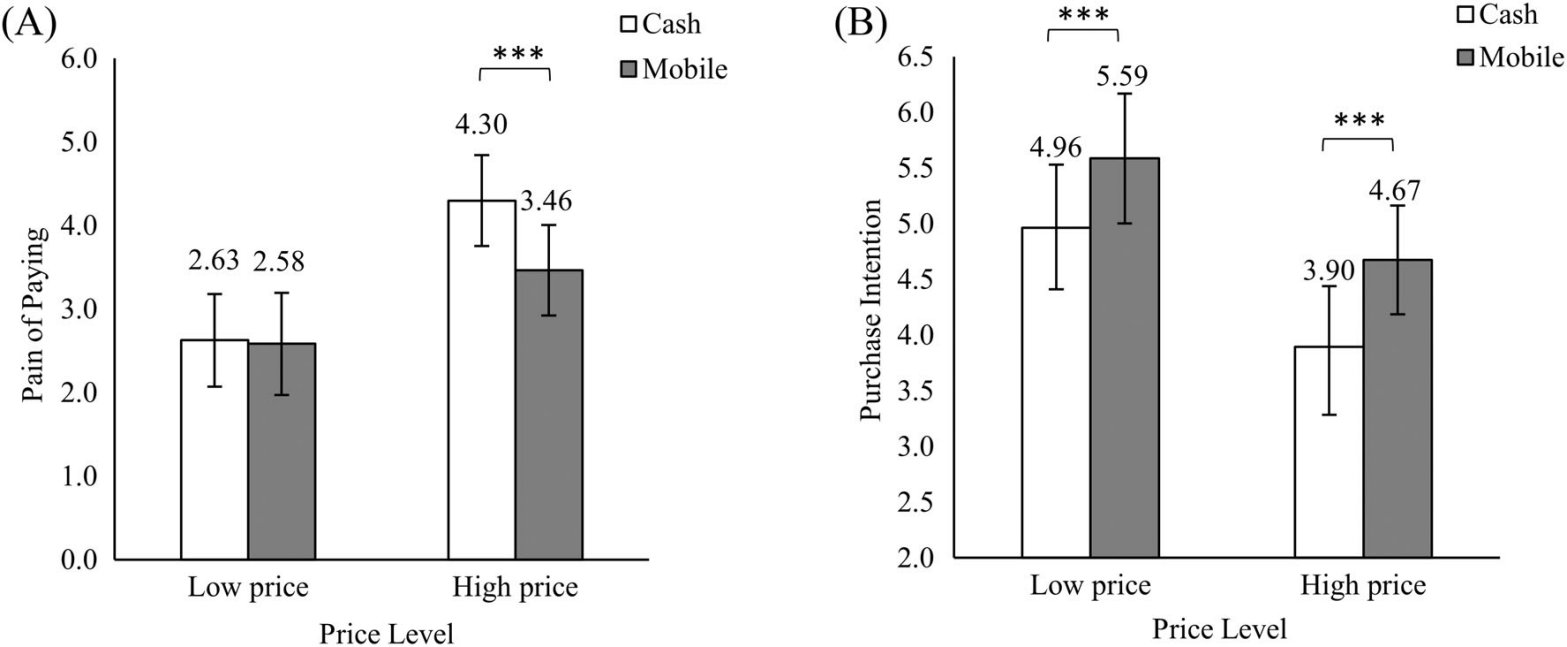
The interactive effect of payment method and price level on (A) pain of paying and (B) purchase intention. Error bars indicate standard deviation. ***p < .001.

Products belonging to the same category had the same prices that we obtained from a pretest. Therefore, for each of the eight categories, we performed a two‐way analysis of variance (ANOVA) with pain of paying as the dependent variable to test H1. Payment method and price level were between‐subjects factors; preference, gender, age, and the frequency of mobile shopping were covariates. The Bonferroni method was used for post hoc tests of significant main effects and interactions (see Supplementary Material C for results of each category). Since six categories (i.e., cookie, shrimp strip, potato chip, beverage, candy, and chocolate) had similar patterns of results, Figure 2A shows the averaged results. There were significant main effects of payment method ($M_{\text{mobile}}$ = 3.023, $M_{\text{cash}}$= 3.461; *F*(1,152) = 26.423, *p <* .001) and price level ($M_{\mathrm{low}}$ = 2.604, $M_{\text{high}}$ = 3.880; *F*(1,152) = 227.081, *p <* .001). Most importantly, there was a significant interactive effect between payment method and price level (*F*(1,152) = 21.016, *p <* .001). That is, mobile payment (vs. cash) significantly reduced pain of paying only in the high‐price condition ($M_{\text{mobile}\_\text{high}}$ = 3.463, $M_{\text{cash}\_\text{high}}$ = 4.300; *F*(1,152) = 48.016, *p <* .001), but not in the low‐price condition ($M_{\text{mobile}\_\mathrm{low}}$ = 2.583, $M_{\text{cash}\_\mathrm{low}}$ = 2.625; *F*_(1,152)_ = 0.151, *p =* .698). Hence, H1 was partially supported. We discussed the reasons for the insignificant interactive effect of pastry and yogurt in Supplementary Material C.

#### Purchase intention

For each category, we performed the same two‐way ANOVA with purchase intention as the dependent variable. The patterns of results were similar for all eight categories (see Supplementary Material D for results of each category), so Figure 2B displays the averaged results. We found significant main effects of payment method ($M_{\text{mobile}}$ = 5.130, $M_{\text{cash}}$ = 4.430; *F*(1,152) = 99.663, *p <* .001) and price level ($M_{\mathrm{low}}$ = 5.275, $M_{\text{high}}$ = 4.284; *F*(1,152) = 191.937, *p <* .001). There was no significant interactive effect (*F*(1,152) = 0.679, *p =* .411) because mobile payment significantly increased purchase intention in both the low‐price and high‐price conditions (low price: $M_{\text{mobile}\_\mathrm{low}}$ = 5.586, $M_{\text{cash}\_\mathrm{low}}$ = 4.964, *F*(1,152) = 41.248, *p <* .001; high price: $M_{\text{mobile}\_\text{high}}$ = 4.673, $M_{\text{cash}\_\text{high}}$ = 3.895, *F*(1,152) = 59.267, *p* < .001), showing a robust mobile payment effect.

#### Moderated mediation results

To test H2, PROCESS Model 8 (Hayes, 2018) was used to conduct moderated mediation analysis, with price level as the moderator and pain of paying as the mediator. We averaged pain of paying and purchase intention across six categories (excluding pastry and yogurt, which were not significant in the analysis about pain of paying). In the regressions, cash payment was coded as 0 and mobile payment as 1; low price was coded as 0 and high price as 1; preference, gender, age, and the frequency of mobile shopping were covariates. With 10,000 bootstrapped resampling, the results revealed a significant moderated mediation effect (index = 0.3883, 95% CI [0.2015, 0.6059]). That is, the mediation effect of pain of paying was significant only in the high‐price condition (indirect effect = 0.4118, 95% CI [0.2294, 0.6205]), but not in the low‐price condition (indirect effect = 0.0235, 95% CI [−0.0704, 0.1360]). Hence, H2 was supported.

### Discussion

Study 1 revealed that mobile payment significantly increased purchase intention regardless of the price level. Furthermore, this behavioral result was mediated by the pain of paying when product prices were relatively high; whereas at low prices, the significant difference in pain between the two payment methods disappeared, thus the mediation effect of pain was not significant. These findings were consistent with H1 and H2.

One finding is worth highlighting: In the low‐price condition, subjects who used mobile payment still reported higher purchase intentions, but the pain of paying was not the underlying mechanism. This implies that there are uncovered mechanisms also driving purchases, and consequently, Study 2 was conducted in a low‐price context to investigate whether the implicit pleasure of payment served as a new psychological mechanism.

## STUDY 2: EYE‐TRACKING EXPERIMENT

Study 2 was conducted with eye‐tracking to test the existence of pleasure of payment (H3 and H4) by examining the consumers' attention to benefits. We used a single‐factor (payment method) within‐subjects design with a time interval of at least 10 days to eliminate interference from memory. The within‐subjects design could exclude the potential influences of variables belonging to individual traits (e.g., eye movement patterns, and mobile shopping frequency). In addition, we used an incentive‐compatible design (i.e., real purchases at the end of the experiment) to enhance the reality for subjects, which could be more effective in eliciting real purchase intentions.

### Participants and design

A total of 62 subjects were recruited from a university in southern China. The reward for participation consisted of a fixed CNY 10.00 and a desired imported food with a net value of CNY 7.00–10.00. Three subjects' data were excluded because they did not pass eye movement calibration, or the percentage of fixation on the target screen^1^ and the percentage of fixation on the area of interests (AOIs)^2^ in most trials were too low to track valid data. As such, data analysis was implemented with data from 59 valid subjects (31 females; mean age = 21.71 years, *SD* = 2.56 years), among which 29 subjects participated in the mobile payment experiment first and then in the cash payment experiment.

Twenty food items and their low prices, which came from the six categories in Study 1, were used as experimental products (see Appendix B) to create the same low‐price context. In terms of the manipulation of payment method, first, we placed either an Alipay icon or a cash payment icon on the desktop within the subject's line of sight (Feinberg, 1986; Liu & Dewitte, 2021). Second, similar to Study 1, subjects were informed that the supermarket only accepted mobile payment or cash payment and watched the corresponding payment video at the beginning of the experiment. Last and most important, subjects used their smartphone or cash to make real payments for a desired food at the end of the experiment.

In terms of product attributes, tastiness and healthfulness were taken as objective attributes as they are the two most salient attributes of food (Dickinson & Kakoschke, 2021; Motoki et al., 2018). Whether an attribute was a benefit or cost depended on its rating. Specifically, both tastiness and healthfulness had five ratings that were similar to the Likert scale: Ratings “1” and “2” represented *poor*, thus belonging to costs; ratings “4” and “5” represented *good*, thus belonging to benefits; and rating “3” meant *neither poor nor good*. According to different combinations of tastiness ratings and healthfulness ratings, products could be classified into three types. As shown in Appendix B, Type‐A was tasty (i.e., benefit) and unhealthy (i.e., cost); Type‐B was healthy (i.e., benefit) and non‐tasty (i.e., cost); Type‐C was other combinations, serving as filler products. There were five Type‐A, five Type‐B, and 10 Type‐C products, and the positions of tastiness and healthfulness on the screen were counterbalanced among product types.

### Apparatus

The experimental equipment consisted of a display screen (for participants) with an SMI RED 250 infrared eye tracker, a computer for making experimental paradigm through the SMI experiment center (for experimenter), a keyboard, and a mouse. While participating in the experiment, subjects were seated ~70 cm from the display screen and used the keyboard and mouse to complete tasks. Eye movement was automatically tracked at a sampling rate of 250 Hz and pre‐processed by BeGaze 3.8 Software.

### Procedure

**FIGURE 3:**
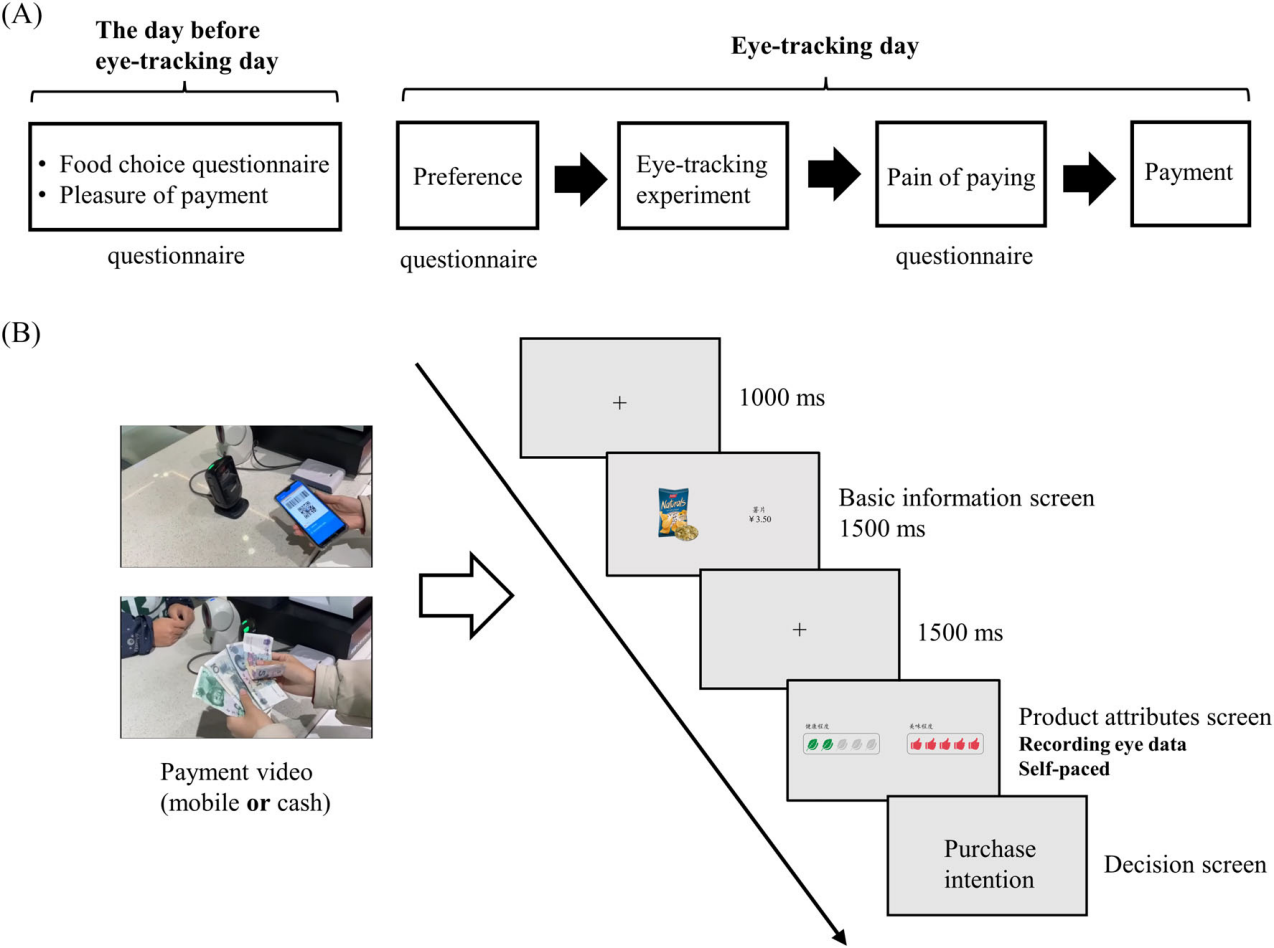
(A) The experimental procedure in Study 2. (B) The paradigm of the eye‐tracking experiment.

The experimental procedure is illustrated in Figure 3A. On the day before the eye‐tracking experiment, subjects completed a food choice questionnaire (FCQ) and a pleasure of payment questionnaire (See Measures for contents). In addition, subjects were told of the only payment method accepted for the next day's shopping trip. On the day of the eye‐tracking experiment, after signing an informed consent form, subjects first evaluated their preferences for each product. Then, we provided a paper cover story whose main content was to elucidate the objectivity of tastiness and healthfulness attributes. Simply speaking, the tastiness rating came from the ranking of sales in the past year, while the healthfulness rating was derived from the ranking of the weighted value of four ingredients content (i.e., protein, fat, carbohydrate, sodium). Subjects were required to consider the attribute information and then evaluate their real purchase intention for each product. After completing the reading, subjects sat in front of the computer and adjusted their sitting posture. The eye tracker calibrated the fixation position through a 9‐point calibration procedure and the calibration was accepted only when errors in both *x*‐ and *y*‐axis were less than 1°. Figure 3B shows the experimental paradigm (modified from the study of Chatterjee & Rose, 2012), starting with a payment video. In each trial, the basic information screen that displayed the picture, category, and price lasted 1500 ms. Next was the product attributes screen showing tastiness and healthfulness, where eye movement data were automatically recorded. This screen was self‐paced because subjects were told to press the F1 button to move to the next decision screen only after they had determined their purchase intention. In the decision screen, subjects used the mouse to select the purchase intention that had been confirmed before. The experiment consisted of three practice trials and 20 randomized formal trials. Finally, a post‐questionnaire measured the subjects' pain of paying for each product. After all of the measurements were completed, we randomly selected a product with high purchase intention (rated 5, 6, or 7), and subjects paid the corresponding price with Alipay or cash to complete the purchase. After at least 10 days, the same experimental procedure was implemented, with the only difference being the payment method.

### Measures

Two non‐overlapping and equally sized AOIs for benefits and costs were defined, and we focused on the subjects' fixation duration on the two AOIs. As the product attributes screen was self‐paced, which meant that stimulus duration total (SDT)^3^ varied among products and subjects, we calculated the percentage‐of‐fixation duration (PFD)^4^ for benefits and costs, respectively. For Type‐A products, tasty was the benefit and unhealthy was the cost; whereas for Type‐B products, healthy was the benefit and non‐tasty was the cost. Type‐C products as filler trials were excluded from the data analysis, and we discussed the reasons and did additional analysis in Supplementary Material F.

Purchase intention was the dependent variable, measured by the same 7‐point Likert scale as in Study 1. The PFD for benefits was the mediator. We also measured other variables: First, sensory appeal factor (four items; e.g., “Tastes good”; α = .742) and health factor (six items; e.g., “Keeps me healthy”; α = .838) were used from an FCQ to measure the importance of tastiness and healthfulness when subjects choose food (Fotopoulos et al., 2009; Steptoe et al., 1995; 1 = *not at all important*, 7 = *very important*). We then computed the FCQ difference (i.e., averaged sensory appeal score minus averaged health score) as a subject‐level variable to capture individual differences (see Supplementary Material E for more details). Second, self‐reported pleasure of payment was assessed by referring to the scale of pain of paying (e.g., “How pleasurable would you feel to pay CNY 3.50 by smartphone/cash when considering purchase intention?”; 1 = *not at all*, 7 = *very pleasurable*), trying to see whether subjects detected implicit mood state. Finally, we measured subjects' preferences and pain of paying for each product using the same 7‐point Likert scale as in Study 1.

### Results

#### An initial test on the pain of paying

To test the effect of payment method on the pain of paying, we averaged the pain of paying for 20 products because they were all at low prices. A paired‐sample *T*‐test was implemented since the difference in pain between mobile and cash payment conditions was normally distributed (K‐S test: *p =* .20 > .05). The results revealed that there was no significant difference in the pain of paying between mobile payment ($M_{\text{mobile}}$ = 3.478) and cash payment ($M_{\text{cash}}$ = 3.442; t(58) = 0.33, *p =* .743), which reconfirms that low price eliminates the difference in pain between the two payment methods.

#### Descriptive results

Table 1 provides descriptive statistics of the attention to each AOI, self‐reported pleasure, and purchase intentions in each payment condition. All data in the table are the original data before standardization.

**TABLE 1:** Descriptive statistics of attention and self‐reported data.

Payment method	PFD for benefits		PFD for costs		Self‐reported pleasure of payment		Purchase intention	
	Mean	*SD*	Mean	*SD*	Mean	*SD*	Mean	*SD*
Mobile	0.3527	0.1336	0.3216	0.1294	4.41	1.401	4.13	1.465
Cash	0.3197	0.1399	0.3263	0.1406	4.21	1.407	3.42	1.523

*Note*: All data in the table are the original data before standardization.

Abbreviations: PFD, percentage‐of‐fixation duration; *SD*, standard deviation.

#### Analysis strategy

There was repeated‐measurement data with a 2 (cash and mobile payment conditions) × 2 (Type‐A and Type‐B products per payment condition) × 5 (five different products per type) within‐subjects design, and we excluded 35 trials in which the PFD for benefits and/or costs were too low. Thus, the main dataset has 1145 records (i.e., 59 subjects × 20 trials – 35 trials). Although we had a relatively small sample size, each participant had almost 20 trials to suffice for statistical power (Baker et al., 2021; Nebe et al., 2023). Given our design, we treated each trial as the fixed effect (Level 1) and nested them within each subject to cater for the random effects (Level 2) in the following analyses.

#### Regression results

The generalized linear mixed‐effects model (GLMM) is suitable for our dataset due to its advantages of handling complicated repeated measures (Anderson et al., 2010; Molenberghs et al., 2010). Therefore, we first employed GLMM via SPSS 26.0 to preliminarily analyze the relationships among payment methods, attention, and purchase intentions.

**Purchase intention.** In the GLMM, purchase intention was the dependent variable; subjects were set as random effects; payment method (mobile vs. cash), product type (A vs. B) and their interactions were fixed effects; preference, pain, gender, age, and FCQ difference were control variables. Consistent with the notion of mobile payment effect, Table 2 shows that mobile payment had a significantly positive influence on purchase intention (β = 0.533, *p < *.001).

**TABLE 2:** Standardized coefficients of generalized linear mixed‐effects model analyses.

Dependent variable	Purchase intention	PFD for benefits	PFD for costs
Intercept	−0.249 (0.689)	−0.155 (0.709)	−0.002 (0.719)
Payment method (cash = 0, mobile = 1)	0.533*** (0.073)	0.293*** (0.0748)	−0.027 (0.079)
Product type (A = 0, B = 1)	0.000 (0.073)	0.040 (0.074)	−0.006 (0.078)
Mobile × Type‐B	−0.120 (0.104)	−0.076 (0.106)	−0.015 (0.111)
Preference	0.105*** (0.032)	0.034 (0.033)	−0.003 (0.034)
Pain	−0.064* (0.032)	−0.048 (0.0328)	0.065 (0.034)
FCQ difference^a^	0.105 (0.055)	0.109 (0.061)	−0.026 (0.052)
Gender (female = 0, male = 1)	−0.013 (0.111)	−0.028 (0.122)	0.042 (0.105)
Age	−0.056 (0.055)	−0.061 (0.060)	0.032 (0.052)
AICc	3131.922	3170.665	3260.522
BIC	3241.788	3280.532	3370.389
N	1145	1145	1145

*Note*: The number in the first row of every cell is the coefficient. The standard error of each coefficient is displayed in parentheses. Significance levels *** *p* < .001, * *p* < .05.

Abbreviations: AICc, Akaike information criterion with a small sample correction; BIC, Bayesian information criterion; FCQ, food choice questionnaire; PFD, percentage‐of‐fixation duration.

^a^ Averaged sensory appeal score minus averaged health score (subject‐level variable).

**The PFD for benefits and costs.** We performed the same GLMM analysis with the PFD for benefits and costs as the dependent variable, respectively. As shown in Table 2, we found that mobile payment (vs. cash) significantly increased subjects' PFD for benefits (*β* = 0.293, *p < *.001), regardless of whether the benefits were tasty or healthy (*β* = −0.706, *p = *.469). However, in terms of costs, there was no significant relationship between the payment method and the PFD for costs (*β* = − 0.027, *p =* .735).

We additionally contrasted the benefits and costs within the payment condition. In the mobile payment condition, the PFD for benefits (*M*_b_ = 35.27%) was significantly higher than that for costs (*M*_c_ = 32.16%; t(565) = 3.203, *p =* .001). In the cash payment condition, there was no significant difference between the PFD for benefits (*M*_b_ = 31.87%) and the PFD for costs (*M*_c_ = 32.63%; t(578) = −0.671, *p = *.502). Altogether, these results showed that mobile payment biased attention toward benefits but cash payment did not, and when compared to cash, mobile payment led to greater attention to benefits.

### Multilevel mediation results

**FIGURE 4:**
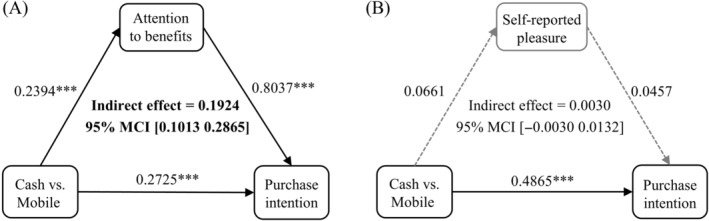
The mediation results of the (A) attention to benefits and (B) self‐reported pleasure in mobile payment effect. ***p < .001.

Based on the results of regressions, we implemented a multilevel mediation analysis via MLmed Macro in SPSS 26.0 (Hayes & Rockwood, 2020; Rockwood, 2017) to further explore the indirect effect of the PFD for benefits. In the model, subjects were set as cluster, product type, preference, and pain as Level‐1 covariates, and FCQ difference, gender, and age as Level‐2 covariates. This model estimates confidence intervals for mediation effects through the Monte Carlo method. Through 20,000 Monte Carlo resampling, we found that the PFD for benefits significantly mediated the promotion effect of mobile payment on purchase intention (see Figure 4A: indirect effect = 0.1924, 95% CI [0.1013, 0.2865]).

Additionally, we conducted a same mediation analysis with the self‐reported pleasure of payment as the mediator (instead of the PFD on benefits). There was no significant mediating effect of the self‐reported pleasure (see Figure 4B: indirect effect = 0.0030, 95% CI [−0.0030 0.0132]), suggesting that the attention to benefits is a more effective indicator of implicit pleasure than subjective rating because consumers had low introspective abilities to subtle implicit mood.

## DISCUSSION

In Study 2, we replicated Study 1's findings of a difference in purchase intention but no difference in pain of paying between cash and mobile payment in the low‐price condition. The result that the cash payment lacked selective attention also supported the absence of pain of paying to some extent. More importantly, by finding that consumers with mobile payment unconsciously deploy their attention to product benefits and also gaze more at benefits than cash payment, we demonstrated the existence of higher pleasure for mobile payment. Hence, H3 was supported. We also further proved the mediating effect of the attention to benefits rather than the self‐reported pleasure, which shows the effectiveness of objective eye‐tracking in revealing implicit mood and supports our H4. In conclusion, Study 2 provides observable evidence for the pleasure of payment as a psychological mechanism behind the mobile payment effect when the pain of paying is not at work.

## GENERAL DISCUSSION

In two experimental studies, we consistently confirmed the behavioral outcome of increased purchase intention with mobile payment versus cash (i.e., mobile payment effect). Moreover, Study 1 showed that the pain of paying mediated the mobile payment effect only in the high‐price condition; whereas for low‐priced products, the difference in pain between the two payment methods was negligible, and the pain of paying failed to explain the increased purchase intention of mobile payment. Then, the pleasure of payment, manifested externally by the spontaneous attention to benefits in Study 2, emerged as the underlying mechanism when the pain of paying was not at play. Additionally, since past mobile shopping frequency is likely to affect the strength of the association between mobile payment and consumption, thereby affecting the role of implicit pleasure, we controlled for this individual variable in both studies, which increases the reliability of our findings.

Crucially, “pleasure of payment” and “pain of paying” are different in nature, and they are not the two ends of a scale. The former is a product of a conditioned learning process, which means that this pleasure exists only after people have a positive experience of using a certain payment method, and it is triggered by the payment cue. The latter, however, is an immediate negative emotion accompanying each expenditure and is directly related to monetary loss, with the payment method being only one of the influencing factors. The three features of mobile payment (fluency, perception of the smartphone, and low pain of paying) contribute to the positive valence of the association, but the pleasure of payment is not constituted by these features because it is born as a new emotion after a positive association has been successfully established.

In sum, Study 2 complements the results of Study 1 well, and the two studies jointly show that the mobile payment effect comes not only from the lower pain of paying but also from the higher pleasure of payment. The pleasure helps us understand consumer behaviors from the perspective of mobile payment increasing motivation to spend (i.e., “stepping on the gas”), which is different from the pain of paying account that claims mobile payment weakens the salience of money loss (i.e., “releasing the brakes”).

### Theoretical implications

Following the calls for research on mobile shopping marketing (Shankar et al., 2016), the present study makes theoretical contributions to the literature on mobile payment and consumer behaviors. We first extend the research on the pain of paying by providing evidence that price level is an important boundary condition for the role of pain of paying in the mobile payment effect. By finding that low prices eliminate the difference in pain between mobile and cash payment, we show that price is a more direct factor influencing the pain of paying than the payment method. The low price means that consumers only lose a quite small amount of money (a small loss in the absolute sense), at which point the difference in transparency of money loss between mobile and cash payment is no longer sufficient to have an impact on the perceived pain. Our results could also advance understanding of why the pain of paying cannot fully explain the increased purchases for cashless payments. For example, Banker et al. (2021) found that a credit card did not reduce pain but increased spending because all products in their experiment were priced at 30% of their retail prices, which created a low‐price context. Moreover, by measuring the pain of paying and validating the interactive effect of payment method and price on pain, our study provides empirical evidence for Shah's (2015) theoretical logic that the choice overload behaviors were affected by the pain of paying.

More importantly, our work provides a thorough discussion of the “pleasure of payment” based on the Cue Theory of Consumption and innovatively understands its role in the mobile payment effect from the perspective of selective attention. Concretely, our extensions of previous research are as follows.

First, while Banker et al. (2021) concluded that credit card (vs. cash) triggered the pursuit of reward by finding stronger activation in the striatum (i.e., a part of the reward network), we further clarify that the nature of this reward is probably a consumption‐related anticipatory hedonic response. Our study hence clearly conceptualizes the implicit hedonic response as “pleasure of payment” and extends it to mobile payment. Moreover, an exploratory electroencephalogram (EEG) study found that the logo of mobile payment first evoked a smaller N300 amplitude and then a greater late positive potential (LPP) amplitude (Wang et al., 2022). It seems that the two components could represent pain and pleasure, respectively, but the LPP could also be a measure of emotion regulation (Hajcak et al., 2010) and thus imply the sequential outcome of a painful emotion rather than a new emotion. Our Study 2 controls the difference in pain between cash and mobile payment and shows powerful evidence for the “pleasure of payment” as a positive driver. The proposed pleasure may also explain Boden et al.'s (2020) finding that mobile payment (vs. credit card) increased WTP only after personal adoption (i.e., a certain level of use frequency). As the two payment methods have a similar extent of pain of paying, one overlooked mechanism at work is the pleasure of payment, an implicit affect based on prior use experiences.

Second, our theorization of pleasure emphasizes the importance of use experiences and association valence, which also contributes to deeply understanding prior literature that manipulated the presence or absence of credit card logos. Some studies showed that mere exposure to credit card logos stimulated spending (Feinberg, 1986; McCall et al., 2004; McCall & Belmont, 1996), while others were not able to replicate this finding (Hunt et al., 1990; Shimp & Moody, 2000), and even found that credit card logos inhibited spending (Lie et al., 2010). This is likely because different experiences of using credit cards have influenced the association with consumption. When participants generally had positive experiences and attitudes with credit cards (Feinberg, 1990; Shimp & Moody, 2000), the credit card built a positive association with consumption and could elicit hedonic responses (pleasure) to promote spending. For those participants who suffered from credit card debt (Hafalir & Loewenstein, 2009; Lie et al., 2010; Shimp & Moody, 2000), however, the credit card could be a negative stimulus that negatively associated with consumption and discouraged spending. For credit card non‐holders (Nakajima & Izumida, 2015), it was simply not a salient cue and did not affect spending. If the effect of a credit card on spending is viewed only from the perspective of the pain of paying (i.e., reducing the salience of money loss), then credit card payment at least should not reduce spending.

In addition to these theoretical contributions, we also show how eye‐tracking technology can advance the understanding of the psychological element behind the mobile payment effect, which enriches research in consumer neuroscience. Cognitive neuroscience methods (e.g., fMRI, EEG, eye‐tracking) can circumvent the self‐report barriers when target mental constructs are implicit and difficult to express (Karmarkar & Plassmann, 2019; Plassmann et al., 2015). The millisecond‐level eye tracking in our study provides an operationalized measure of the implicit pleasure of payment by tracking consumers' attention in real‐time. The results that self‐reported pleasure failed to predict purchases also highlight the superiority of eye‐tracking in revealing intrinsic emotions and cognitions.

### Practical implications

The findings of this study have practical implications for consumers, merchants, and payment‐service providers. First, our research shows that consumers' increased purchases with mobile payment are driven by unconscious pleasure in addition to lower immediate pain. From the perspective of maximizing consumer welfare, we suggest consumers increase payment steps by canceling password‐free payments and shifting attention from the recreational applications to the payment process, which could mitigate the positive associations between mobile payment and consumption.

Second, merchants should realize that mobile payment is not just a tool for completing transactions; it can affect consumers' shopping experience and increase sales. We recommend merchants promote mobile payment in retail environments as much as possible, such as equipping with self‐checkout devices, which shorten payment time and increase consumers' engagement and control over payments. Supermarket chains can launch shopping applications with payment functions (e.g., Walmart Pay in the Walmart app) and issue coupons to encourage consumers to embrace mobile payment. These measures will be helpful to strengthen the pleasure of payment and broaden sales channels. Based on our eye‐tracking results, we also advise merchants to put the positive attributes of products in the spotlight by using bright labels (e.g., an exaggerated “Hot Sale” label) or other means, aiming to attract consumers' first attention to products. When coupled with the greater attention that mobile payment directs, the labels will bring more purchases.

Finally, we suggest that payment‐service providers should optimize the payment process, such as simplifying the interaction interface to increase processing fluency (Orth & Wirtz, 2014) and introducing more fluent forms of payment (e.g., face payments) and more portable payment devices (e.g., wearable payments). Service providers can also give additional benefits (e.g., green energy and payment points) after consumers complete each transaction. These measures will increase the overall utility of the shopping journey and then enhance the pleasure of payment.

### Limitations and future directions

This study has some limitations. First, it was carried out in China, where cash payment directly crossed to mobile payment; however, the dominant cashless payment method in some Western countries is still the credit card. Although mobile payment has more characteristics of building positive associations with consumption, Western consumers have longer use experiences of credit cards. Future research can collect data from Western countries to validate the pleasure of payment and explore its effect size by comparing the credit card with mobile payment. Also, focusing on consumers who are in debt due to their credit card may find more interesting results.

Second, as a pioneering work to test the pleasure of payment with eye tracking, we conducted Study 2 in the low‐price context, aiming to provide reliable evidence independent of the pain of paying. This is a relatively conservative approach, which at the same time brings limitations to the current study. Although the pleasure of payment is shown to be at work in the low‐price condition in this study, it could be generalized for all products as long as people have prior positive experiences of using mobile payment, as the pleasure is a product of the conditioned learning process (Laibson, 2001). Considering that the pleasure of payment is theoretically a universal psychological mechanism and the pain of paying mechanism has been shown not to work for low‐priced products, we believe that a more interesting future direction, in addition to increasing the generalizability of the pleasure of payment, is to focus on the relationship between these two mechanisms. To be more specific, future research could focus on when they work together and when one of the mechanisms plays a dominant role. According to the Person‐Situation Interactions theory (Funder, 2006) that helps to understand “why people do what they do”, the coexistence and dominance between the two psychological mechanisms may depend on individual and situational factors and their interaction. Individual factors refer to personal traits. For example, researchers have shown that individuals differ in their sensitivity to spending, measured by the Tightwad–Spendthrift Scale (Rick et al., 2008). Such individual differences may enhance or weaken the pain of paying mechanism. In terms of the situational factors, in addition to the price level demonstrated in this study (i.e., low prices weaken the pain of paying, making implicit pleasure dominant), sensory stimulation from mobile devices is also a factor worth exploring. Manshad and Brannon (2021) have found that low‐intensity vibration feedback from smartphones can increase the pain of paying; whereas multisensory stimulation can facilitate the learning process (Shams & Seitz, 2008), which may strengthen the positive associations between mobile payment and consumption.

At the methodological level, fMRI may be a suitable tool to explore the above issues because all emotions are closely related to specific structures and systems in the brain. Given that the mesolimbic dopamine system is thought to be involved in the process of cue‐triggered pleasure and desire (Bernheim & Rangel, 2004), and the activation of anterior insula has been shown to characterize pain of paying (Mazar et al., 2016), fMRI allows researchers to further explore the neural basis of implicit pleasure and its relationship with the pain of paying, so as to enrich the research on mobile payment.

## CONFLICT OF INTEREST STATEMENT

The authors declare there are no conflicts of interest.

## ETHICS STATEMENT

All procedures performed in studies involving human participants were approved by and in accordance with the ethical standards of the Ethics Committee of Neuromanagement Laboratory of Zhejiang University and with the 1964 Helsinki Declaration and its later amendments or comparable ethical standards. Informed consent was obtained from all participants included in the study.

## Supporting information


**Data S1.** Supporting information.

## Data Availability

The data that support the findings of this study are available from the corresponding author upon reasonable request.

## Endnotes

^1^
The target screen refers to the product attributes screen, where subjects' eye movements were recorded. Percentage of fixation on the target screen = $\frac{\text{Fixation duration}\;\mathrm{on}\;\text{the target screen}\;\left(\mathrm{ms}\right)}{\text{Stimulus duration}\;\text{total}\;\mathrm{on}\;\text{the target}\;\text{screen}\;\left(\mathrm{ms}\right)}$. Stimulus duration total (SDT) is the total time a screen appears, and it is equal to the end time minus the start time.

^2^
“AOIs” here refers to two non‐overlapping and equally sized areas for benefits and costs; the percentage of fixation on an AOI = $\frac{\text{Fixation duration}\;\mathrm{on}\;\text{an}\;\mathrm{AOI}\;\left(\mathrm{ms}\right)}{\text{Fixation duration}\;\mathrm{on}\;\text{target screen}\;\left(\mathrm{ms}\right)}$.

^3^
Stimulus duration total (SDT) is the total time a screen appears, and it is equal to the end time minus the start time.

^4^
Percentage of fixation duration (PFD) = $\frac{\text{Fixation duration}\;\mathrm{on}\;\text{an}\;\mathrm{AOI}\;\left(\mathrm{ms}\right)}{\text{Stimulus duration total}\;\mathrm{on}\;\text{the product attributes screen}\;\left(\mathrm{ms}\right)}$.

## APPENDIX A Experimental materials in Study 1

**Table:**

	Category	Picture	Low price	High price
Cookie and Pastries	Cookie		CNY 3	CNY 8
			CNY 3	CNY 8
	Pastry		CNY 5	CNY 9
			CNY 5	CNY 9
Puffed food	Shrimp strip		CNY 3	CNY 7.5
	Potato chip		CNY 3.5	CNY 9
			CNY 3.5	CNY 9
Drinks	Beverage		CNY 4	CNY 8
			CNY 4	CNY 8
	Yogurt		CNY 6	CNY 15
			CNY 6	CNY 15
Sugar food	Candy		CNY 4	CNY 9
			CNY 4	CNY 9
	Chocolate		CNY 4.5	CNY 10
			CNY 4.5	CNY 10

## APPENDIX B Experimental materials in Study 2

### B.1. Type‐A products in Study 2

**Table:**

Category	Basic information screen	Product attributes screen
Cookie		
Shrimp strip		
Potato chip		
Beverage		
Candy		

### B.2. Type‐B products in Study 2

**Table:**

Category	Basic information screen	Product attributes screen
Cookie		
Potato chip		
Beverage		
Candy		
Chocolate		

### B.3. Type‐C products in Study 2

**Table:**

Category	Basic information screen	Product attributes screen
Cookie		
Potato chip		
Beverage		
Chocolate		
Cookie		
Potato chip		
Beverage		
Candy		
Cookie		
Potato chip		
