# Supplementary material for: Why does mobile payment promote purchases? Revisiting the pain of paying, and understanding the implicit pleasure via selective attention
Source: Psych J. 2024 May 16;13(5):760–79. doi: 10.1002/pchj.765 (PMC11444724; doi:10.1002/pchj.765)
Supplement: Supplementary file 1 — Data S1. Supporting information. [file PCHJ-13-760-s001.docx]

# Supplementary materials about Study 1

## The self-report measure on pain of paying in previous studies

The pain of paying is defined as an explicit negative emotion from the (anticipated) act of giving money and thus is directly related to the financial loss (see Reshadi & Fitzgerald, 2023, for a review), By emphasizing “spending a specific amount of money” or “paying a certain amount of bills” in the questionnaire, people are guided to think about and capture the negative feelings associated with money losses. In this way, a large number of previous studies have directly asked participants about their perceived pain of paying, and we compiled only a part of these studies in Table S1. These studies with diverse consumption scenarios and experimental products (diverse prices including low prices, e.g., Soster et al., 2014) were published in high-quality journals, and the samples were from multiple countries, covering multiple age groups and social status.

**Table S1** Previous studies (part) using scale to measure pain of paying.

| **Article** | **Studies (n, location)** | **Pain of paying scale** | **Payment methods** |
| --- | --- | --- | --- |
| Thomas et al. (2011) | Study 3 (n = 124, US) | “How did you feel about spending money on this shopping trip?”  *(5-point Facial emoticon scale)* | Cash  Credit card |
| Shah et al. (2016) | Study 1 (n = 63, US) | “How painful was paying for the mug when you originally bought it?”  *(7-point Likert scale)* | Cash  Cards |
|  | Study 3 (n = 189, US) | “How painful was paying for the headphones (i.e., how painful was giving up your money)?”  *(5-point Likert scale)* | Cash  Debit card |
| Boden et al. (2020) | Study 1 (n = 250, India, US) | “How painful would it feel to pay...”  *(7-point Likert scale)* | Cash  Credit card  Mobile payment |
|  | Study 2 (n = 294, India, Germany) |  |  |
|  | Study 3 (n = 204, US) |  |  |

**Table S1** **(continued)** Previous studies (part) using scale to measure pain of paying.

| **Article** | **Studies (n, location)** | **Pain of paying scale** | **Payment methods** |
| --- | --- | --- | --- |
| H. Liu & Chou (2020) | Study 1 (n = 154, China) | “I would feel pain if I decided to pay for [exchange] the product with the money [store points]”  *(7-point Likert scale)* | Cash  Store points |
|  | Study 3 (n = 60, China) |  | Cash  Gift certificates |
| Y. Liu & Dewitte (2021) | Study 2 (n = 205, US) | “How did you feel about spending money on this shopping trip?”  *(5-point Facial emoticon scale)* | Cash  Credit card  Mobile payment |
|  | Study 3 (n = 118, Belgium) |  |  |
|  | Study 4 (n = 191, Belgium) |  |  |
| Manshad & Brannon (2021) | Pretest (n = 60, US) | “To what extent did the payment feel expensive? to what extent did the payment make you think about the cost of the item? to what extent did the payment make you think about losing money”  *(7-point Likert scale)* | Mobile payment |
| Park et al. (2021) | Study 1 (n = 104, US) | “How did you feel about spending money with cash [card]?”  *(5-point Facial emoticon scale)* | Cash  Cards |
|  | Study 2 (n = 401, US) |  |  |
| Wang et al. (2022) | Study 1 (n = 66, China) | “How painful did you feel when making this purchase decision with cash [Alipay]?”  *(7-point Likert scale)* | Cash  Mobile payment |
| Zellermayer, (1996) | Study 1 (n = 34, US) | “How painful it is when pay a bill….”  *(11-point Likert scale)* | - |
|  | Study 2 (n = 120, US) |  | - |
| Rick et al. (2008) | Study 2 (n = 1087, US) | “How painful would it be to pay for the massage?”  *(7-point Likert scale)* | - |
| Soster et al. (2014) | Study 3 (n = 194, US) | “How did you feel about spending 10 credits to purchase this film?”  *(5-point Facial emoticon scale)* | - |
|  | Study 4 (n = 106, US) |  | - |
|  | Study 5 (n = 291, US) | “How did you feel about spending $2 to purchase this film?”  *(5-point Facial emoticon scale)* | - |

## Mobile shopping frequency

The frequency of mobile shopping was measured with reference to Hou & Elliott (2021). Subjects were asked that “On average, how many times do you use smartphones for shopping in a day?”. Table S2 presents descriptive statistics on the mobile shopping frequency for subjects in the different payment conditions.

**Table S2** Descriptive statistics for the mobile shopping frequency in Study 1.

| **Payment condition** | **Frequency of mobile shopping** | **Percentage** |
| --- | --- | --- |
| Mobile payment  (n = 80) | 0 times a day | 1.25% |
|  | 1-3 times a day | 68.75% |
|  | 4-6 times a day | 18.75% |
|  | 7-9 times a day | 5.00% |
|  | 10 or more times a day | 6.25% |
| Cash payment  (n = 80) | 0 times a day | 2.50% |
|  | 1-3 times a day | 65.00% |
|  | 4-6 times a day | 2.25% |
|  | 7-9 times a day | 6.25% |
|  | 10 or more times a day | 3.75% |

## Analysis of variance (ANOVA) results on pain of paying for each product category

For each of the eight categories, we conducted a 2 (payment method: cash payment vs. mobile payment) × 2 (price level: low price vs. high price) between-subjects ANOVA on pain of paying, with preference, gender and age as covariates. The Bonferroni method was used to perform post hoc tests for significant main and interaction effects. The reason why we did not directly average across all categories is that the overall averaged result reflects a central tendency, which is not convincing when the pattern of results is not similar among categories.

As shown in Table S3, only six of the eight categories had a similar pattern of results: A significant main effect of payment method, with higher pain of paying for cash payment; a significant main effect of price level, with higher pain of paying for high-priced products; and a significant interactive effect, with the pain of mobile payment being lower than that of cash payment only in the high-price condition.

For the Pastry, no significant interactive effect was observed (*F*(1,152) = 0.245, *p* = 0.621). This was because the pain of mobile payment was lower than that of cash payment in both low-price and high-price conditions (see Table S3 and Figure S1a), suggesting that the low price for the Pastry (CNY 5.00) was not low enough to dominate the pain of paying. In addition, for the Yogurt, neither the main effect of payment method (*F*(1,152) = 1.498, *p* = 0.223) nor the interactive effect (*F*(1,152) = 0.063, *p* = 0.0803) was significant. This was due to no difference in pain between the two payment methods in both low-price and high-price conditions (see Table S3 and Figure S1b), suggesting that the high price for the Yogurt (CNY 15.00) was not high enough and therefore led to the same effect as low price.

**Table S3** The ANOVA results of payment method and price level on pain of paying for each food category.

| Category | Main effect of payment method | Main effect of price level | Interaction effect | Simple effect | |
| --- | --- | --- | --- | --- | --- |
| Cookie | $M_{mobile}$= 2.944 $M_{cash}$= 3.356 *F*(1,152) =7.480  *p*=0.007 | $M_{low}$=2.513 $M_{high}$=3.788 *F*(1,152)=69.515  *p*=0.000 | $M_{mobile\_low}$=2.538 $M_{mobile\_high}$=3.350 $M_{cash\_low}$=2.488 $M_{cash\_high}$=4.225 *F*(1,152)=9.168  *p*=0.003 | high-price: *F*(1,152)=16.887  *p*=0.000 | low-price: *F*(1,152)=0.044  *p*=0.835 |
| Pastry | $M_{mobile}$=3.106 $M_{cash}$=3.794 *F*(1,152)=22.428  *p*=0.000 | $M_{low}$=2.750 $M_{high}$=4.150 *F*(1,152)=79.321  *p*=0.000 | $M_{mobile\_low}$=2.463 $M_{mobile\_high}$=3.750 $M_{cash\_low}$=3.037 $M_{cash\_high}$=4.550 *F*(1,152)=0.223  *p*=0.637 | high-price: *F*(1,152)=13.794  *p*=0.000 | low-price: *F*(1,152)=8.928  *p*=0.003 |

**Table S3 (continued)** The ANOVA results of payment method and price level on pain of paying for each food category.

| Category | Main effect of payment method | Main effect of price level | Interaction effect | Simple effect |  |
| --- | --- | --- | --- | --- | --- |
| Shrimp strip | $M_{mobile}$=3.170 $M_{cash}$=3.660 *F*(1,152)=7.206  *p*=0.008 | $M_{low}$=2.690 $M_{high}$=4.150 *F*(1,152)=74.952  *p*=0.000 | $M_{mobile\_low}$=2.650 $M_{mobile\_high}$=3.700 $M_{cash\_low}$=2.730 $M_{cash\_high}$=4.600 *F*(1,152)=5.715  *p*=0.018 | high-price: *F*(1,152)=13.096  *p*=0.000 | low-price: *F*(1,152)=0.042  *p*=0.837 |
| Potato chip | $M_{mobile}$=3.187 $M_{cash}$=3.669 *F*(1,152)=10.506  *p*=0.001 | $M_{low}$=2.725 $M_{high}$=4.131 *F*(1,152)=104.761  *p*=0.000 | $M_{mobile\_low}$=2.700 $M_{mobile\_high}$=3.675 $M_{cash\_low}$=2.750 $M_{cash\_high}$=4.587 *F*(1,152)=10.267  *p*=0.002 | high-price: *F*(1,152)=21.149  *p*=0.000 | low-price: *F*(1,152)=0.001  *p*=0.976 |
| Beverage | $M_{mobile}$=3.056 $M_{cash}$=3.506 *F*(1,152)=11.251  *p*=0.001 | $M_{low}$=2.763 $M_{high}$=3.800 *F*(1,152)=51.655  *p*=0.000 | $M_{mobile\_low}$=2.737 $M_{mobile\_high}$=3.375 $M_{cash\_low}$=2.787 $M_{cash\_high}$=4.225 *F*(1,152)=5.978  *p*=0.016 | high-price: *F*(1,152)=17.082  *p*=0.000 | low-price: *F*(1,152)=0.405  *p*=0.525 |
| Yogurt | $M_{mobile}$=2.481 $M_{cash}$=2.638 *F*(1,152)=1.595  *p*=0.209 | $M_{low}$=2.144 $M_{high}$=2.975 *F*(1,152)=43.661  *p*=0.000 | $M_{mobile\_low}$=2.088 $M_{mobile\_high}$=2.875 $M_{cash\_low}$=2.200 $M_{cash\_high}$=3.075 *F*(1,152)=0.044  *p*=0.835 | high-price: *F*(1,152)=1.101  *p*=0.296 | low-price: *F*(1,152)=0.546  *p*=0.461 |
| Candy | $M_{mobile}$=2.988 $M_{cash}$=3.487 *F*(1,152)=12.701  *p*=0.000 | $M_{low}$=2.638 $M_{high}$=3.838 *F*(1,152)=83.399  *p*=0.000 | $M_{mobile\_low}$=2.625 $M_{mobile\_high}$=3.35 $M_{cash\_low}$=2.650 $M_{cash\_high}$=4.325 *F*(1,152)=12.774  *p*=0.000 | high-price: *F*(1,152)=25.915  *p*=0.000 | low-price: *F*(1,152)=0.000  *p*=0.995 |
| Chocolate | $M_{mobile}$=2.788 $M_{cash}$=3.325 *F*(1,152)=12.884  *p*=0.000 | $M_{low}$=2.300 $M_{high}$=3.813 *F*(1,152)=108.788  *p*=0.000 | $M_{mobile\_low}$=2.250 $M_{mobile\_high}$=3.325 $M_{cash\_low}$=2.350 $M_{cash\_high}$=4.300 *F*(1,152)=9.939  *p*=0.002 | high-price: *F*(1,152)=23.129  *p*=0.000 | low-price: *F*(1,152)=0.093  *p*=0.761 |


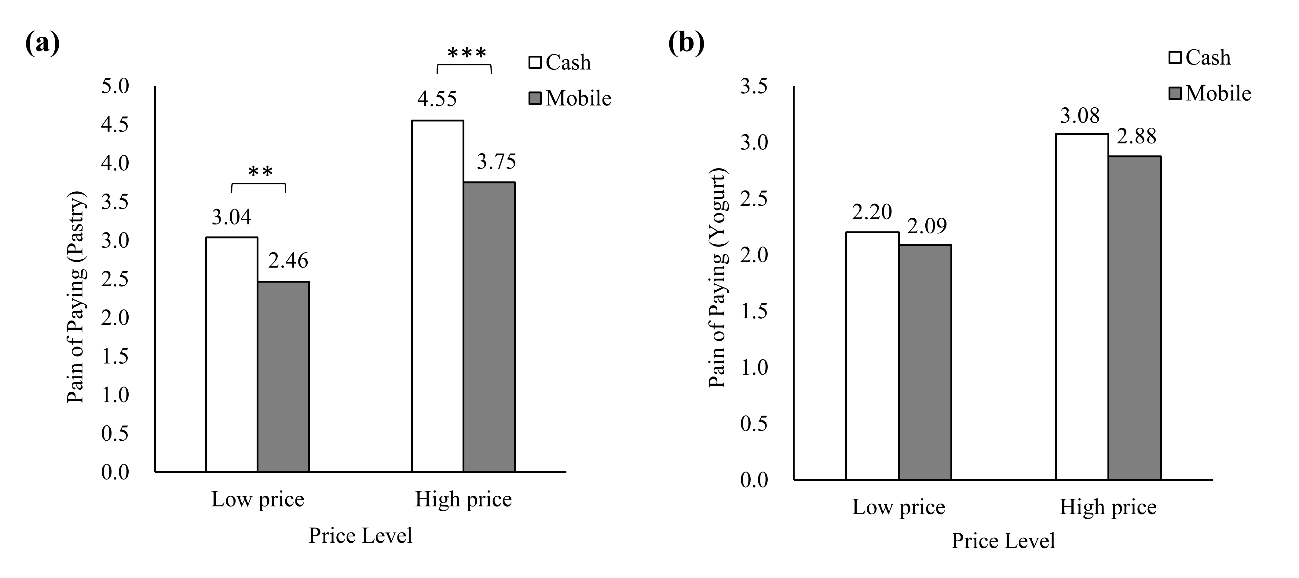


**Figure S1** The interactive effect of payment method and price level on pain of paying for **(a)** Pastry and **(b)** Yogurt. **p*<0.05, ***p*<0.01, ****p*<0.001.

## ANOVA results on purchase intention for each product category

Similarly, for each of the eight categories, we conducted a 2 (payment method: cash payment vs. mobile payment) × 2 (price level: low price vs. high price) between-subjects ANOVA on purchase intention, with preference, gender and age as covariates. The Bonferroni method was used to perform post hoc tests for significant main and interaction effects.

As showed in Table S4, all categories had similar results: A significant main effect of payment method, with higher purchase intention for mobile payment; a significant main effect of price level, with higher purchase intention for low-priced products; and an insignificant interactive effect, as mobile payment increased purchase intention in both low-price and high-price conditions.

**Table S4** The ANOVA results of payment method and price level on purchase intention for each food category.

| Category | Main effect of payment method | Main effect of price level | Interaction effect | Simple effect | |
| --- | --- | --- | --- | --- | --- |
| Cookie | $M_{mobile}$=5.237 $M_{cash}$=4.481 *F*(1,152)=31.152  *p*=0.000 | $M_{low}$=5.356 $M_{high}$=4.363 *F*(1,152)=43.283  *p*=0.000 | $M_{mobile\_low}$=5.700 $M_{mobile\_high}$=4.775 $M_{cash\_low}$=5.013 $M_{cash\_high}$=3.950 *F*(1,152)=0.017  *p*=0.886 | high-price: *F*(1,152)=16.557  *p*=0.000 | low-price: *F*(1,152)=14.596  *p*=0.000 |
| Pastry | $M_{mobile}$=5.000 $M_{cash}$=4.300 *F*(1,152)=29.809  *p*=0.000 | $M_{low}$=5.206 $M_{high}$=4.094 *F*(1,152)=62.468  *p*=0.000 | $M_{mobile\_low}$=5.462 $M_{mobile\_high}$=4.538  $M_{cash\_low}$=4.950 $M_{cash\_high}$=3.650 *F*(1,152)=1.286  *p*=0.259 | high-price: *F*(1,152)=22.114  *p*=0.000 | low-price: *F*(1,152)=9.189  *p*=0.003 |
| Shrimp strip | $M_{mobile}$=4.890 $M_{cash}$=4.100 *F*(1,152)=16.781  *p*=0.000 | $M_{low}$=5.130 $M_{high}$=3.860 *F*(1,152)=51.100  *p*=0.000 | $M_{mobile\_low}$=5.500 $M_{mobile\_high}$=4.270 $M_{cash\_low}$=4.750 $M_{cash\_high}$=3.450 *F*(1,152)=0.130  *p*=0.719 | high-price: *F*(1,152)=10.098  *p*=0.002 | low-price: *F*(1,152)=6.865  *p*=0.010 |
| Potato chip | $M_{mobile}$=4.962 $M_{cash}$=4.313 *F*(1,152)=17.486  *p*=0.000 | $M_{low}$=5.094 $M_{high}$=4.181 *F*(1,152)=37.932  *p*=0.000 | $M_{mobile\_low}$=5.375 $M_{mobile\_high}$=4.550 $M_{cash\_low}$=4.813 $M_{cash\_high}$=3.812 *F*(1,152)=0.489  *p*=0.485 | high-price: *F*(1,152)=12.141  *p*=0.001 | low-price: *F*(1,152)=5.971  *p*=0.016 |
| Beverage | $M_{mobile}$=5.062 $M_{cash}$=4.381 *F*(1,152)=35.574  *p*=0.000 | $M_{low}$=5.441 $M_{high}$=4.300 *F*(1,152)=45.748  *p*=0.000 | $M_{mobile\_low}$=5.438 $M_{mobile\_high}$=4.688 $M_{cash\_low}$=4.850 $M_{cash\_high}$=3.913 *F*(1,152)=0.001  *p*=0.970 | high-price: *F*(1,152)=18.289  *p*=0.000 | low-price: *F*(1,152)=17.282  *p*=0.000 |
| Yogurt | $M_{mobile}$=5.606 $M_{cash}$=5.063 *F*(1,152)=24.439  *p*=0.000 | $M_{low}$=5.706 $M_{high}$=4.962 *F*(1,152)=32.211  *p*=0.000 | $M_{mobile\_low}$=5.950 $M_{mobile\_high}$=5.262 $M_{cash\_low}$=5.463 $M_{cash\_high}$=4.662 *F*(1,152)=0.019  *p*=0.891 | high-price: *F*(1,152)=13.122  *p*=0.000 | low-price: *F(*1,152)=11.348  *p*=0.001 |

**Table S4 (continued)** The ANOVA results of payment method and price level on purchase intention for each food category.

| Category | Main effect of payment method | Main effect of price level | Interaction effect | Simple effect |  |
| --- | --- | --- | --- | --- | --- |
| Candy | $M_{mobile}$=5.094 $M_{cash}$=4.356 *F*(1,152)=31.498  *p*=0.000 | $M_{low}$=5.206 $M_{high}$=4.244 *F*(1,152)=64.470  *p*=0.000 | $M_{mobile\_low}$=5.538 $M_{mobile\_high}$=4.65 $M_{cash\_low}$=5.000 $M_{cash\_high}$=3.887 *F*(1,152)=0.422  *p*=0.517 | high-price: *F*(1,152)=19.953  *p*=0.000 | low-price: *F*(1,152)=12.116  *p*=0.001 |
| Chocolate | $M_{mobile}$=5.188 $M_{cash}$4.444 *F*(1,152)=21.946  *p*=0.000 | $M_{low}$=5.362 $M_{high}$=4.269 *F*(1,152)=47.830  *p*=0.000 | $M_{mobile\_low}$=5.725 $M_{mobile\_high}$=4.650 $M_{cash\_low}$=5.000 $M_{cash\_high}$=3.887 *F*(1,152)=0.021  *p*=0.886 | high-price: *F*(1,152)=11.856  *p*=0.001 | low-price: *F*(1,152)=10.130  *p*=0.002 |

# Supplementary materials about Study 2

## Data processing of Food Choice Questionnaire

We used the sensory appeal factor and the health factor from the Food Choice Questionnaire (FCQ; Steptoe et al., 1995) to measure the importance of tastiness and healthfulness in subjects’ food choices (1 = not at all, 7 = very important; Fotopoulos et al., 2009). The four items of sensory appeal factor are “Smells nice”, “Looks nice”, “Has a pleasant texture” and “Tastes good” ($\alpha$ = 0.742). The six items of health factor are “Contains a lot of vitamins and minerals”, “Keeps me healthy”, “Is nutritious”, “Is high in protein”, “Is good for my skin/teeth/hair/nails etc.” and “Is high in fiber and roughage” ($\alpha$ = 0.838).

A total of 59 subjects completed FCQ on the day before each eye-tracking experiment (i.e., they completed FCQ twice with a time interval of at least 10 days). Since the difference values between two measurements of sensory appeal factor did not obey a normal distribution (K-S test: *p* = 0.009 < 0.05), we implemented the Wilcoxon signed-rank test, which is a nonparametric test for paired samples. The results showed that there was no significant difference between the two measurements of sensory appeal factor ($M_{SAtime1}$ = 5.640, $M_{SAtime2}$ = 5.593; Z = $-$0.387, *p* = 0.699). In addition, the difference values between two measurements of health factor obeyed a normal distribution (K-S test: *p* = 0.20 > 0.05). The paired sample T-test results also showed no significant difference between the two measurements of health factor ($M_{Htime1}$ = 4.718, $M_{Htime2}$ = 4.703; $t_{Htime1-Htime2}(58)$ = 0.168, *p* = 0.867). The above results indicate that the sensory appeal factor and the health factor indeed measure individuals’ motivation when choosing food in daily life and that the two factors belong to stable individual traits. Therefore, for each subject, we computed the averaged sensory appeal score and the averaged health score. Then, their difference value (i.e., averaged sensory appeal score minus averaged health score) could measure the relative importance between tastiness and healthfulness, which entered into our eye data analyses as a subject-level variable.

## Additional analysis on Type-C products

The Type-C products as filler trials were excluded from the data analysis for the following two reasons: First, although the rating of “3” in Type-C products essentially meant neither poor nor good (i.e., no bias), the cognition and classification criteria of this rating were likely to vary among subjects. In other words, whether subjects tended to perceive the rating of “3” as a benefit or a cost or no bias depended on whether it reached the lower limit of their requirements for a food. Second, the vast majority of products sold in real life have good and bad attributes in objective and/or subjective aspects, which is why consumers need to make trade-offs in their decisions. There may indeed be completely good and completely poor products, but the differences between payment methods are probably not enough to have an observable effect on purchase decisions of such products due to little tangle and trade-off in decisions. We confirmed this conjecture by analyzing the differences in decision time and purchase intention across payment methods and product types.

To explore whether subjects exhibit decision polarization for Type-C products, decision time on the benefit-cost screen (i.e., Stimulus Duration Total; SDT) and purchase intention were focused. First, we conducted a 2 (payment method: mobile payment vs. cash payment) × 3 (product type: A vs. B vs. C) repeated-measure ANOVA for the SDT of benefit-cost screen. If the sphericity assumption is violated, the significance results are Pillai's Trace values of the multivariate tests. The Bonferroni method was used for post hoc tests of significant main effects and interactions. We found a significant main effect of product type (*F*(2,57) = 3.585, *p* = 0.034; $M_{A}$ = 4512.581, $M_{B}$ = 4639.591, $M_{C}$ = 4289.824). Further pairwise comparisons revealed that the SDT of Type-B products was significantly higher than that of Type-C products (*p* = 0.029), as shown in Figure S2a. Neither a significant main effect of payment method (*F*(1,58) = 0.308, *p* = 0.581) nor a significant interactive effect (*F*(2,116) = 0.245, *p* = 0.783) was observed.

Second, in terms of the purchase intention, we divided Type-C products into good (C1) and poor (C2) subtypes according to the overall ratings on tastiness and healthfulness. Then, a 2 (payment method: mobile payment vs. cash payment) × 4 (product type: A vs. B vs. C1 vs. C2) repeated-measure ANOVA was conducted. The Bonferroni method was used for post hoc tests of significant main effects and interactions. As shown in Figure S2b, we found a significant main effect of payment method (*F*(1,58) = 23.324, *p* < 0.001; $M_{mobile}$ = 4.106, $M_{cash}$ = 3.712) and a significant main effect of product type (*F*(3,174) = 134.373, *p* < 0.001; $M_{A}$ = 3.782, $M_{B}$ = 3.731, $M_{C1}$ = 5.278, $M_{C2}$ = 2.846). Further pairwise comparisons indicated that Subtype-C1 and Subtype-C2 had the significantly highest (*p* < 0.001) and lowest (*p* < 0.001) purchase intentions, respectively. We also observed a significant interactive effect (*F*(3,174) = 11.584, *p* < 0.001), that is, mobile payment increased purchase intention for Type-A and Type-B products; whereas payment method did not affect purchase intention for Subtype-C1 and Subtype-C2 products.

In sum, compared to Type-A and Type-B products, subjects’ decisions about Type-C products had a lower degree of entanglement and trade-offs, which was reflected in less decision time and more polarized purchase intentions. In other words, the difference in pleasure of payment between mobile and cash payment might be not powerful enough to influence the polarized purchase intention of Type-C products.


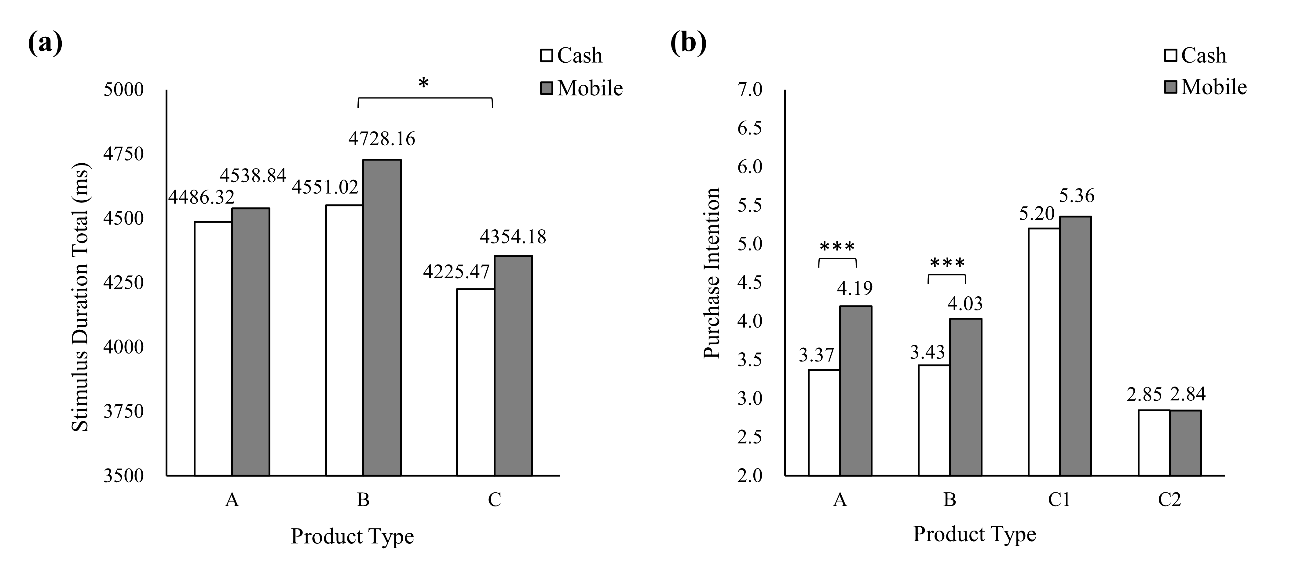


**Figure S2** The interactive effect of payment method and product type on **(a)** stimulus duration total and **(b)** purchase intention. *p<0.05, **p<0.01, ***p<0.001.

# References

Boden, J., Maier, E., & Wilken, R. (2020). The effect of credit card versus mobile payment on convenience and consumers’ willingness to pay. *Journal of Retailing and Consumer Services*, *52*, 101910. https://doi.org/10.1016/j.jretconser.2019.101910

Fotopoulos, C., Krystallis, A., Vassallo, M., & Pagiaslis, A. (2009). Food Choice Questionnaire (FCQ) revisited. Suggestions for the development of an enhanced general food motivation model. *Appetite*, *52*(1), 199–208. https://doi.org/10.1016/j.appet.2008.09.014

Hou, J., & Elliott, K. (2021). Mobile shopping intensity: Consumer demographics and motivations. *Journal of Retailing and Consumer Services*, *63*, 102741. https://doi.org/10.1016/j.jretconser.2021.102741

Liu, H., & Chou, H. (2020). Payment formats and hedonic consumption. *Psychology & Marketing*, *37*(11), 1586–1600. https://doi.org/10.1002/mar.21404

Liu, Y., & Dewitte, S. (2021). A replication study of the credit card effect on spending behavior and an extension to mobile payments. *Journal of Retailing and Consumer Services*, *60*, 102472. https://doi.org/10.1016/j.jretconser.2021.102472

Manshad, M. S., & Brannon, D. (2021). Haptic-payment: Exploring vibration feedback as a means of reducing overspending in mobile payment. *Journal of Business Research*, *122*, 88–96. https://doi.org/10.1016/j.jbusres.2020.08.049

Park, J., Lee, C., & Thomas, M. (2021). Why Do Cashless Payments Increase Unhealthy Consumption? The Decision-Risk Inattention Hypothesis. *Journal of the Association for Consumer Research*, *6*(1), 21–32. https://doi.org/10.1086/710251

Reshadi, F., & Fitzgerald, M. P. (2023). The pain of payment: A review and research agenda. *Psychology & Marketing*, *40*(8), 1672–1688. https://doi.org/10.1002/mar.21825

Rick, S. I., Cryder, C. E., & Loewenstein, G. (2008). Tightwads and Spendthrifts. *Journal of Consumer Research*, *34*(6), 767–782. https://doi.org/10.1086/523285

Shah, A. M., Eisenkraft, N., Bettman, J. R., & Chartrand, T. L. (2016). “Paper or Plastic?”: How We Pay Influences Post-Transaction Connection. *Journal of Consumer Research*, *42*(5), 688–708. https://doi.org/10.1093/jcr/ucv056

Soster, R. L., Gershoff, A. D., & Bearden, W. O. (2014). The Bottom Dollar Effect: The Influence of Spending to Zero on Pain of Payment and Satisfaction. *Journal of Consumer Research*, *41*(3), 656–677. https://doi.org/10.1086/677223

Steptoe, A., Pollard, T. M., & Wardle, J. (1995). Development of a Measure of the Motives Underlying the Selection of Food: The Food Choice Questionnaire. *Appetite*, *25*(3), 267–284. https://doi.org/10.1006/appe.1995.0061

Thomas, M., Desai, K. K., & Seenivasan, S. (2011). How Credit Card Payments Increase Unhealthy Food Purchases: Visceral Regulation of Vices. *Journal of Consumer Research*, *38*(1), 126–139. https://doi.org/10.1086/657331

Wang, M., Ling, A., He, Y., Tan, Y., Zhang, L., Chang, Z., & Ma, Q. (2022). Pleasure of paying when using mobile payment: Evidence from EEG studies. *Frontiers in Psychology*, *13*, 1004068. https://doi.org/10.3389/fpsyg.2022.1004068

Zellermayer, O. (1996). *The Pain of Paying* [Doctoral dissertation]. Carnegie Mellon University.
